# Supplementary material for: Zinc utilisation, trends, and predictors among under-five children with diarrhoea in Ethiopia: A pooled analysis
Source: PLoS One. 2024 Nov 19;19(11):e0314127. doi: 10.1371/journal.pone.0314127 (PMC11575758; doi:10.1371/journal.pone.0314127)
Supplement: Attachment — Submitted filename: Review comment 11.docx [file pone.0314127.s001.docx]

- The manuscript does not meet the standards of written English. We suggest having the paper edited by language professionals or native speakers.
- In Table 1, the author classified regions as developed and undeveloped. What criteria did the author use to categorize the country's regions into these groups? The author needs to justify the criteria.
- The author must construct tables appropriately; for example, table 2 is not constructed correctly.
- The authors have to ensure that the methodology section clearly explains the response and covariates of the study.
- The author needs to provide a more detailed discussion of their findings and compare and contrast them with the existing literature in the study's discussion section.
- Major concern: The author considers DHS data from 2005-2016, but it's worth noting if they observe the trend of zinc utilization, which I didn't see in the paper.
- If the authors addressed the issues mentioned above, I would recommend accepting and publishing the manuscript after revision.
