# Supplementary material for: Zinc utilisation, trends, and predictors among under-five children with diarrhoea in Ethiopia: A pooled analysis
Source: PLoS One. 2024 Nov 19;19(11):e0314127. doi: 10.1371/journal.pone.0314127 (PMC11575758; doi:10.1371/journal.pone.0314127)
Supplement: Attachment — Submitted filename: Response to reviewer_Zinc utilisation_R1.docx [file pone.0314127.s002.docx]

Zinc utilisation, trends, and predictors among under-five children with diarrhoea in Ethiopia: a pooled data analysis: PONE-D-24-39627

The author would like to acknowledge the Editor and reviewers for giving me an opportunity to revise the manuscript.

**Response to editor**: The author made changes to the manuscript.
Reviewers' comments:

Reviewer #1: The paper is clearly structured and the topic of this research is very interesting to
address the public health problem of diarrhea and to show policy gaps on zinc utilization.
1. In the introduction, lines 51-55 describe the utilization of zinc and its predictors. What are your research gaps? Clearly state the research gap in the introduction section
after line 55.

**Authors’ response**: The author made changes to the introduction section.
2. Check line 75; statement of size using the 2005-2019 primary health care data. However, you were using EDHS data from 2005-2016.

**Authors’ response**: The author made changes to the methods section.
3. Write some statement about exclusion criteria?

**Authors’ response**: The author made changes to the methods section.
4. Would you consistently use the term "under five" or "under-five" throughout your
document?

**Authors’ response:** The author made changes to the manuscript.
5. How do you select study participants from urban and rural residences to avoid
geographical effects?

**Authors’ response**: The author made changes to the manuscript.
6. What is your study population: under-five children with mothers, or under-five
children with diarrhea and their mothers? Please write clearly.

**Authors’ response**: The author made changes to the manuscript.
7. Write briefly on the MOR value like =1 or ≥1. indicates?

**Authors’ response**: The author made changes to the methods section.
8. On lines 152-153, check whether the AOR is within the 95% CI, and also verify
this against the table.

**Authors’ response**: The author made changes to the results section.
9. On limitation, line 189, you stated that there is limited literature. However, other
references are available in Africa, including Burundi, Comoros, Kenya, Ethiopia, Madagascar, Malawi, Tanzania, Uganda, and Zimbabwe. References
1. Seifu BL, Legesse BT, Yehuala TZ, Kase BF, Asmare ZA, Mulaw GF, Tebeje
TM, Mare KU. Factors associated with the co-utilization of oral rehydration
solution and zinc for treating diarrhea among under-five children in 35 sub- saharan Africa countries: a generalized linear mixed effect modeling with robust error variance. BMC Public Health. 2024 May 16;24(1):1329. 2. Kassa SF, Alemu TG, Techane MA, Wubneh CA, Assimamaw NT, Mulualem G, et al. The co-utilization of oral rehydration solution and zinc for treating Diarrhea and its Associated factors among under-five children in Ethiopia: further analysis of EDHS 2016 the co-utilization of oral. Rehydration Solution and Zinc for Treating Diarrhea; 2022.
3. Yimenu DK, Kasahun AE, Chane M, Getachew Y, Manaye B, Kifle ZD. Assessment of knowledge, attitude, and practice of child caregivers towards oral rehydration salt and zinc for the treatment of diarrhea in under 5 children in Gondar town. Clin Epidemiol Glob Heal [Internet]. 2022;14:100998. 4. Ayele E, Tasew H, Mariye T, Teklay G, Alemayhu T, Mesfin F. 2017. Medicine. 2020; 4(1):15–9. 5. Ugwu J, Ezeagu I, Ibegbu M. Awareness and practice of zinc therapy in diarrheal
management among under-five caregivers in Enugu State, Nigeria. International
Journal of Medicine and Health Development. 2019; 24(2):63–9
6. Ajayi D.T BO, Ijaola T.T. Oke O.A. and Fabiyi G.A. Determinants of Oral
Rehydration Solution and Zinc Use Among Under-Five Children for The
Management of Diarrhea in Abeokuta, Nigeria. Archives of Basic and Applied
Medicine 2019; 7:35–9. 7. Emmanuel Firima BF. Knowledge and recommendation of oral rehydration solution and zinc for management of childhood diarrhoea among patent and
proprietary medicine vendors in Port Harcourt, Nigeria. Journal of Global Health
Reports. 2020; 4:1–12
**Authors’ response**: The author made changes to the discussion section as requested.
10. Add other limitation on the nature of the EDHS survey does not include data on
dietary intake of zinc (dietary sources of zinc) which affect the utilization. Sometimes
women do not give zinc tablets because they think that food rich in zinc is a substitute
of zinc tablets.

**Authors’ response**: The author made changes to the discussion section.
11. Discuss the trends in zinc utilization from 2005 to 2016 and explain why zinc
utilization was higher in 2005 compared to 2011 and why there was a sharp increase
in zinc utilization in the 2016 EDHS data.

**Authors’ response**: The author made changes to the discussion section.
12. Conclusion: The prevalence of zinc utilization is low. Compared to what? Write
briefly by comparing with the global or national recommendations.

**Authors’ response**: The author made changes to the conclusion section.
13. Discussion were shallow and revised this section by adding updated references.

**Authors’ response**: The author has updated the discussion section.
14. Your justification on line 170 regarding the difference between socioeconomic
status and cultural status was not convincing. Both studies use EDHS data and are
from the same country? How do you see?

**Authors’ response**: The author expunged the cultural status for clarity purposes.

Reviewer #2: • The manuscript does not meet the standards of written English. We suggest having the paper edited by language professionals or native speakers.

**Authors’ response**: The entire manuscript was thoroughly edited.
• In Table 1, the author classified regions as developed and undeveloped. What criteria did the author use to categorize the country's regions into these groups? The author needs to justify the criteria.

**Authors’ response**: Ethiopia's classification into developed and emerging regions is primarily based on its administrative divisions and economic performance.

**References**: 1.Tamirat KS, Kebede FB, Gonete TZ, Tessema GA, Tessema ZT. Geographical variations and determinants of iron and folic acid supplementation during pregnancy in Ethiopia: analysis of 2019 mini demographic and health survey. BMC Pregnancy Childbirth. 2022;22(1):127. Epub 2022/02/17. doi: 10.1186/s12884-022-04461-0.

2. Gebremedhin T, Geberu DM, Atnafu A. Less than one-fifth of the mothers practised exclusive breastfeeding in the emerging regions of Ethiopia: a multilevel analysis of the 2016 Ethiopian demographic and health survey. BMC Public Health. 2021;21(1):18. Epub 2021/01/06. doi: 10.1186/s12889-020-10071-2.

3. Governent of Ethiopia, Ministry of Federal affairs, emerging regions deveopment programmes: http://efaidnbmnnnibpcajpcglclefindmkaj/https://info.undp.org/docs/pdc/Documents/ETH/00047309_Emerging%20Regions%20Development%20Programme%20(00056833).pdf. 4. <https://www.worldbank.org/en/country/ethiopia/overview>,
• The author must construct tables appropriately; for example, table 2 is not constructed correctly.

**Authors’ response**: The author amended tables.
• The authors have to ensure that the methodology section clearly explains the response and covariates of the study.

**Authors’ response**: The author made changes to the methods section. The study variables were operationalised based on the existing evidence.
• The author needs to provide a more detailed discussion of their findings and compare and contrast them with the existing literature in the study's discussion section.

**Authors’ response**: The authors made changes to the discussion section.
• Major concern: The author considers DHS data from 2005-2016, but it's worth noting if they observe the trend of zinc utilization, which I didn't see in the paper.

**Authors’ response**: The authors made changes to the discussion section.
