# Supplementary material for: Zinc utilisation, trends, and predictors among under-five children with diarrhoea in Ethiopia: A pooled analysis
Source: PLoS One. 2024 Nov 19;19(11):e0314127. doi: 10.1371/journal.pone.0314127 (PMC11575758; doi:10.1371/journal.pone.0314127)
Supplement: Attachment — Submitted filename: Response to Reviewers_Zinc utilization_Rv2.docx [file pone.0314127.s003.docx]

Zinc utilisation, trends, and predictors among under-five children with diarrhoea in Ethiopia: a pooled analysis: PONE-D-24-39627R1

The author thanks to the Editor and reviewers for giving me an opportunity to revise the manuscript.

**Response to editor**: The author revised the manuscript.
Reviewers' comments:

Reviewer #1: The authors should be attentive while editing the tables. In line 169, AOR was not found within the 95% CI; please also check Table 2.

**Author’s response**: The author made changes to the results section.

Revise the discussion sections in lines 233-238 and 241-242 based on your findings. You are discussing training and cost-related activities, though your findings were not revealed on training and cost.

**Author’s response**: The author expunged it for clarity purposes.
